# Research on atrial fibrillation mechanisms and prediction of therapeutic prospects: focus on the autonomic nervous system upstream pathways

**DOI:** 10.3389/fcvm.2023.1270452

**Published:** 2023-11-08

**Authors:** Jingjie Huang, Bangqi Wu, Peng Qin, Yupei Cheng, Ziyi Zhang, Yameng Chen

**Affiliations:** ^1^ Postgraduate School, Tianjin University of Traditional Chinese Medicine, Tianjin, China; ^2^ National Clinical Research Center for Chinese Medicine Acupuncture and Moxibustion, First Teaching Hospital of Tianjin University of Traditional Chinese Medicine, Tianjin, China

**Keywords:** atrial fibrillation, stroke, therapies, mechanism, forecast

## Abstract

Atrial fibrillation (AF) is the most common clinical arrhythmia disorder. It can easily lead to complications such as thromboembolism, palpitations, dizziness, angina, heart failure, and stroke. The disability and mortality rates associated with AF are extremely high, significantly affecting the quality of life and work of patients. With the deepening of research into the brain-heart connection, the link between AF and stroke has become increasingly evident. AF is now categorized as either Known Atrial Fibrillation (KAF) or Atrial Fibrillation Detected After Stroke (AFDAS), with stroke as the baseline. This article, through a literature review, briefly summarizes the current pathogenesis of KAF and AFDAS, as well as the status of their clinical pharmacological and non-pharmacological treatments. It has been found that the existing treatments for KAF and AFDAS have limited efficacy and are often associated with significant adverse reactions and a risk of recurrence. Moreover, most drugs and treatment methods tend to focus on a single mechanism pathway. For example, drugs targeting ion channels primarily modulate ion channels and have relatively limited impact on other pathways. This limitation underscores the need to break away from the “one disease, one target, one drug/measurement” dogma for the development of innovative treatments, promoting both drug and non-drug therapies and significantly improving the quality of clinical treatment. With the increasing refinement of the overall mechanisms of KAF and AFDAS, a deeper exploration of physiological pathology, and comprehensive research on the brain-heart relationship, it is imperative to shift from long-term symptom management to more precise and optimized treatment methods that are effective for almost all patients. We anticipate that drugs or non-drug therapies targeting the central nervous system and upstream pathways can guide the simultaneous treatment of multiple downstream pathways in AF, thereby becoming a new breakthrough in AF treatment research.

## 1. Introduction

Atrial Fibrillation (AF) is the most prevalent clinical arrhythmia, characterized by irregular atrial activity and subsequent loss of mechanical function, making it the most severe atrial electrical activity disorder. The atrium loses its regular and orderly electrical activity, replaced by rapid and disorganized fibrillation waves (1). According to estimates from the 2010 Global Burden of Disease Study, the worldwide prevalence of AF is approximately 33 million people. The incidence of AF is higher in males, while females tend to have a higher mortality rate associated with AF. In countries such as Australia, Europe, and the USA, the prevalence of AF among adults ranges from 1% to 4%, but this figure rises to over 13% among individuals aged 80 and older. In the United States alone, there are an estimated 3–5 million people living with AF. With the aging of the population, it is projected that over 8 million people in the USA will be affected by AF by 2050. In Europe, the prevalence of AF is expected to increase from the current estimated 8.8 million to approximately 18 million by 2060. It is estimated that Japan has around 700,000 individuals with AF, and this number is projected to surpass 1 million by 2050. In China, approximately 3.9 million individuals aged 60 years or older have AF. However, by 2050, as China’s population of individuals aged 60 years or older grows to 460 million, it is estimated that 9 million of them will have AF (2). In 2020, the domestic cardiovascular health and disease report conducted random sampling statistics of community residents in 2015. It found that the prevalence of AF among Chinese residents aged 35 and above was 0.7%, while it was 1.2% for rural Chinese residents aged 35 and above. Within this group, the prevalence was 0.1% for individuals aged 35–44 and 4.6% for those aged at least 75. The prevalence of AF did not differ significantly between genders (3). The most common comorbidities in AF patients include hypertension, followed by coronary heart disease and heart failure. Patients aged over 75 with AF are more likely to have coronary heart disease, hypertension, stroke, cognitive impairment, and chronic obstructive pulmonary disease (COPD).

Every year, there are 8 million cases of ischemic stroke worldwide. Among these, 20% of individuals have prevalent atrial fibrillation, a condition characterized by irregular heart rhythms. In the remaining 80% of patients without known atrial fibrillation (KAF), up to 24% can be newly diagnosed with atrial fibrillation after undergoing long-term electrocardiogram monitoring. Despite this, a significant number of cases still go undiagnosed due to inadequate monitoring (4). As research into the brain-heart axis and brain-heart syndromes continues to expand, scholars have unveiled a close relationship between the brain and the heart. Within this context, stroke-heart syndrome has emerged as a crucial branch of brain-heart research. While some studies suggest there may not be a direct causal relationship between stroke and atrial fibrillation, certain research findings indicate that stroke can act as both a cause and a consequence of atrial fibrillation, or that their interaction may involve more complex mechanisms (5–7). Consequently, an increasing number of studies have started to investigate the interplay between stroke and AF, categorizing AF into two groups: known atrial fibrillation (KAF) and newly discovered atrial fibrillation after stroke (AFDAS), using stroke as a baseline. KAF is primarily driven by structural changes in the heart, and therefore, it can be predominantly considered “cardiogenic.” On the other hand, AFDAS is primarily linked to stroke and may be regarded as “neurogenic,” or a combination of both.

## 2. Mechanisms of KAF

KAF is primarily cardiogenic, often stemming from underlying cardiac abnormalities, and has undergone extensive research (8). These mechanisms encompass atrial remodeling, altered autonomic function, changes in calcium channels and gap junctional proteins, inflammatory responses, and abnormal gene expression. When these underlying mechanisms trigger atrial fibrillation, the rapid and irregular activation of the atria during atrial fibrillation leads to electrical remodeling. This results in the shortening of the atrial refractory period and promotes reentry, creating a detrimental cycle known as “AF begets AF" (9, 10). AF is both a cause and a consequence of atrial heart disease. The pathogenesis of KAF is intricate. In this paper, we will provide a concise overview of the following six aspects (11):

• Electrical remodeling and structural remodeling
• Alterations in the autonomic nervous system
• Calcium-handling remodeling
• Gap-junction remodeling
• Inflammatory responses
• Abnormalities in gene expression

### 2.1. Atrial remodeling

Atrial remodeling primarily encompasses structural remodeling and electrical remodeling. Structural remodeling is recognized as a significant factor in the initiation and persistence of AF (12). It involves changes such as atrial enlargement, cardiomyocyte hypertrophy (13), depolarization, and atrial fibrosis. Research has indicated that fibrosis is frequently observed in AF patients, and the increased presence of fibroblasts, myofibroblasts, and elevated extracellular matrix deposition in fibrotic tissue disrupts the continuity of myocardial bundles and interferes with the gap junctions between cardiomyocytes. Endomyocardial biopsies in patients with isolated atrial fibrillation have revealed abnormal alterations, including myolysis, glycogen accumulation, mitochondrial changes, and signs of chromatin structure depolarization. These changes are characterized by the dispersion and disappearance of the sarcoplasmic reticulum, as well as degeneration and necrosis of atrial myocytes (14). In the right atrium of patients with persistent AF, micronodular content was found to be reduced, and myolysis with loss of cellular myogenic fiber structure was observed (15). Additionally, studies have shown that the activation of the renin-angiotensin-aldosterone system (RAAS), particularly angiotensin II (Ang-II), also contributes to structural remodeling of the atria (16).

Electrical remodeling in AF pertains to alterations in electrophysiological properties induced by AF and is considered a compensatory mechanism to prevent intracellular Ca2+ overload. Calcium overload is implicated in the electrical and structural remodeling of the atria, leading to atrial fibrillation, impairment of cardiac cell vitality, and contractile dysfunction (12, 17). Yoo et al., using a novel gene therapy approach in a canine model of rapid atrial pacing, demonstrated that oxidative damage caused by NADPH oxidase 2 (NOX2) results in the upregulation of acetylcholine-dependent K-current (IKACh) activity. This mechanism is not only the origin but also a perpetuator of electrical remodeling in AF. Experimental evidence also indicated that rapid pacing of canine atrial myocytes is induced by oxidative damage through the induction of NOX2 and the production of mitochondrial reactive oxygen species. This suggests that oxidative damage may trigger electrical remodeling in AF by a mechanism involving the activation of protein kinase C epsilon, causing an upregulation of IKACh (18). Recent studies have also suggested the involvement of SK channels in atrial remodeling in experimental AF models. Cardiac SK channels functionally connect voltage-gated calcium ion channels and are activated during contraction, participating in cardiac action potential (AP) repolarization. Experimental research demonstrated that SK channel inhibition can have antiarrhythmic effects by directly blocking SK channels (19, 20). Channels in the K2P family, such as TWIK-1, TASK-1, and TASK-3, are background potassium channels, and research suggests that they can influence the duration of depolarization in ventricular muscle cells, potentially inducing arrhythmias. Upregulation of K2P currents can lead to APD shortening in chronic AF patients, and inhibition of channels like TWIK-1, TASK-1, and TASK-3 may potentially reverse AF-related APD shortening, thereby inhibiting atrial fibrillation occurrence and preventing electrical remodeling (21, 22).

### 2.2. Changes in autonomic function

Many animal and clinical studies have shown that imbalances in the cardiac autonomic nervous system (ANS) play a vital role in developing and maintaining AF, and that parasympathetic and sympathetic overactivity increase vulnerability to AF (23). The autonomic nervous system communicates extensively with the heart through external inputs and ganglionated plexi (GP) located on the epicardial surface (24). The ANS is closely associated with heart rate variability, and Agarwal et al. noted that impaired cardiac autonomic function, characterized by reduced resting heart rate variability, is associated with a higher incidence of AF (25). Activation of the autonomic nervous system can induce atrial tachyarrhythmias, including atrial tachycardia and AF, by inducing significant and heterogeneous changes in atrial electrophysiology (23). Research indicates that autonomic regulation has a significant impact on cardiac ion channels. Simultaneous activation of the sympathetic and parasympathetic nervous systems, for example, can lead to increased intracellular Ca2+ transients by the sympathetic nervous system and activation of IKAch by the parasympathetic nervous system, resulting in shortened APD (action potential duration) and larger and longer Ca2+ transients. Shortened APD and larger Ca2+ transients create conditions for early afterdepolarizations, which can trigger triggered activity and AF (26). Patterson et al. found that in canine pulmonary veins, rapid discharges and atrial fibrillation could be triggered by simultaneous stimulation of the parasympathetic and sympathetic nervous systems (27). Other studies have suggested that the autonomic nervous system not only contributes to the substrate for AF in normal hearts but also participates in the genesis of structural heart disease, with the parasympathetic system contributing to the maintenance of AF and the sympathetic system affecting the frequency characteristics of AF (28). As demonstrated by Arora, interactions between the vagus nerve and sympathetic nerve stimulation can create ectopic foci, making the autonomic nervous system a trigger for AF. Additionally, in structural heart disease, ANS forms a substrate for AF maintenance (29). Park et al. (30) experimentally demonstrated in a canine model that simultaneous sympathetic discharge was the most common trigger for paroxysmal tachycardia and AF. Chen et al. discussed the importance of autonomic nervous system activity in inducing PAF. They demonstrated that vagal denervation can enhance the efficacy of circumferential pulmonary vein isolation in preventing AF recurrence. Through heart rate variability analysis, they found that sympathetic and parasympathetic imbalances existed before the onset of PAF; sympathetic and vagal discharges occurred simultaneously prior to PAF onset in experimental animals (31). Gould et al. also suggested in their study of persistent AF patients that autonomic remodeling may be a part of the atrial substrate for AF (32). Zhang et al. (33) showed that the vagus nerve can regulate AF through the α7nAChR-mediated cholinergic anti-inflammatory pathway. To our knowledge, the sympathetic nervous system is typically associated with the adrenergic system, while the parasympathetic nervous system is typically associated with the cholinergic system. In the human body, the autonomic nervous system and the adrenergic/cholinergic systems interact to maintain balance. When the sympathetic nervous system is stimulated excessively or abnormally, it can lead to excessive release of adrenaline. Workman stated in their research that adrenergic stimulation by catecholamines can lead to AF in patients. Catecholamines can influence every electrophysiological mechanism of AF initiation and maintenance in human atria (34). In summary, the autonomic nerves inherent to the heart can act as the sole trigger for initiating AF. Furthermore, current research suggests that the autonomic nervous system is linked to other fundamental mechanisms of AF. Modulating the ANS through electrical stimulation has been considered a promising therapeutic strategy in clinical and research settings.

### 2.3. Calcium-handling remodeling

Intracellular calcium (Ca2+) overload and abnormal Ca2+ handling processes can contribute to the development and persistence of AF. Research conducted in animal models and human cardiomyocytes isolated from atrial appendages has revealed that reduced mRNA and protein expression of L-type Ca2+ channels, along with altered phosphorylation and redox potential, result in decreased Ca2+ current density. A reduction in Ca2+ current density is a hallmark of AF (35). Chelu and colleagues discovered that abnormal ryanodine receptor 2 (RyR2) with enhanced calcium sensitivity in a mouse model of AF leads to excessive intracellular calcium release from cardiac myocytes. This, in turn, causes increased activity of calmodulin-dependent protein kinase II (CaMKII), which is a critical downstream effect in individuals susceptible to AF. In a comparison of patients with and without AF, Hove-Madsen et al. found that the development of AF was associated with increased spontaneous calcium release from the sarcoplasmic reticulum in atrial myocytes (36). Some studies have identified significant differences in electrical remodeling and calcium handling among different forms of primary atrial fibrillation (AF), such as paroxysmal AF (pAF), persistent AF (cAF), and persistent AF with heart failure (HFrEF-cAF). Their research demonstrated that abnormal Ca2+ handling promotes ectopic (triggered) activity and reentry through action potential duration (APD) shortening and heterogeneous conduction. These mechanisms are the primary causes of arrhythmia. Classic indicators of atrial electrical remodeling associated with AF primarily appear in cAF and HFrEF-cAF. Ca2+-dependent triggered activity forms the basis for atrial arrhythmias in pAF patients, primarily due to increased sarcoplasmic reticulum (SR) Ca2+ load and dysregulation of RyR2, leading to an increased incidence of spontaneous Ca2+ events (SCaEs), resulting in delayed afterdepolarizations (DAD) and triggered activity. Spontaneous cell activity in central atrial cardiomyocytes increases in pAF patients. In cAF patients’ atrial muscle cells, higher spontaneous Ca2+ release events are observed, along with electrical remodeling characterized by APD shortening and membrane potential hyperpolarization, which promotes reentry. In cAF, the increased SR Ca2+ release is a result of increased RyR2 channel open probability (RyR2 Po) mediated by CaMKII due to excessive phosphorylation of RyR2, making RyR2 channels more sensitive to Ca2+. However, in pAF, there is primarily an increase in SR Ca2+ uptake, opposite to the decrease observed in cAF, and no increase in RyR2 phosphorylation is found, nor is atrial fibrillation-related electrical remodeling observed in pAF cardiomyocytes. Studies in HFrEF-cAF patients did not observe a decrease in connexin-43, but markers of fibrosis (collagen-1a, fibronectin, periostin) were expressed at higher levels. Myosin and RyR2 protein levels both decreased, but SERCA2a expression increased, leading to increased RyR2-Ser2814 phosphorylation, making RyR2 more sensitive to Ca2+ (10, 37, 38).

### 2.4. Gap-junction remodeling

Gap junctions represent a significant determinant of electrical impulse conduction in cardiac tissue (39). In the atria, the primary gap junction subunits are connexins40 and connexins43. Notably, there is substantial heterogeneity in the distribution of connexins throughout different regions of the heart. In a study on goats, it was discovered that the “gap junction remodeling” process is involved in the stabilization of atrial fibrillation. As the duration of atrial fibrillation increases, apart from the redistribution of Cx40, the overall levels of these gap junction proteins also significantly decrease (40). van der Velden et al. in their research indicated that local variations in the expression of connexin proteins (Cx40 or Cx43), whether upregulated or downregulated, may underlie conduction velocity heterogeneity (or dispersion), thereby creating conditions conducive to micro-reentry, which could lead to sustained atrial fibrillation (41). Therefore, Changes in connexins in AF may contribute to local conduction abnormalities and may facilitate the initiation and perpetuation of AF (17).

### 2.5. Inflammatory response

Inflammation and the associated immune response play a role in initiating and sustaining AF. Inflammatory signaling pathways are causally involved in the development of atrial electrical remodeling, calcium handling, and structural remodeling. These pathways can also influence neural growth and autonomic variations by affecting the heart’s intrinsic cardiac nervous system (42). Research has demonstrated that AF can further exacerbate inflammation, and mediators of the inflammatory response can modify atrial electrophysiology and structural substrates, increasing susceptibility to AF. Postoperative atrial fibrillation (POAF) is a common complication following cardiac surgery, often peaking 2–4 days after the procedure. It typically manifests as newly developed atrial fibrillation immediately after surgery, with episodes being short in duration, paroxysmal, and asymptomatic. Evidence suggests that inflammation may be one of the complex mechanisms contributing to the occurrence of POAF. It is associated with inflammatory biomarkers such as IL-2, IL-6, and C-reactive protein (43). In their study, Fakuade and colleagues also found that abnormal calcium handling, primarily impaired SR calcium uptake, not only results in pre-existing atrial contractile dysfunction but also creates a substrate for atrial arrhythmias, making patients more susceptible to POAF (44). Subsequent research by Heijman and others revealed that patients who develop POAF exhibit clear abnormalities in calcium handling and activation of the NLRP3-inflammatory/CaMKII signaling pathway in atrial cardiomyocytes. These molecular substrates render cardiomyocytes sensitive to spontaneous calcium release and arrhythmogenic afterdepolarizations, particularly when exposed to inflammatory mediators (45). Furthermore, inflammation also regulates calcium homeostasis and connexins, which are linked to AF triggers and heterogeneous atrial conduction. Inflammatory pathways mediate myolysis, myocardial apoptosis, and fibrosis through fibroblast activation, transforming growth factor-β signaling, and matrix metalloproteinase activation, all contributing to structural remodeling of the atria (46, 47).

### 2.6. Abnormal gene expression

**Figure 1:**
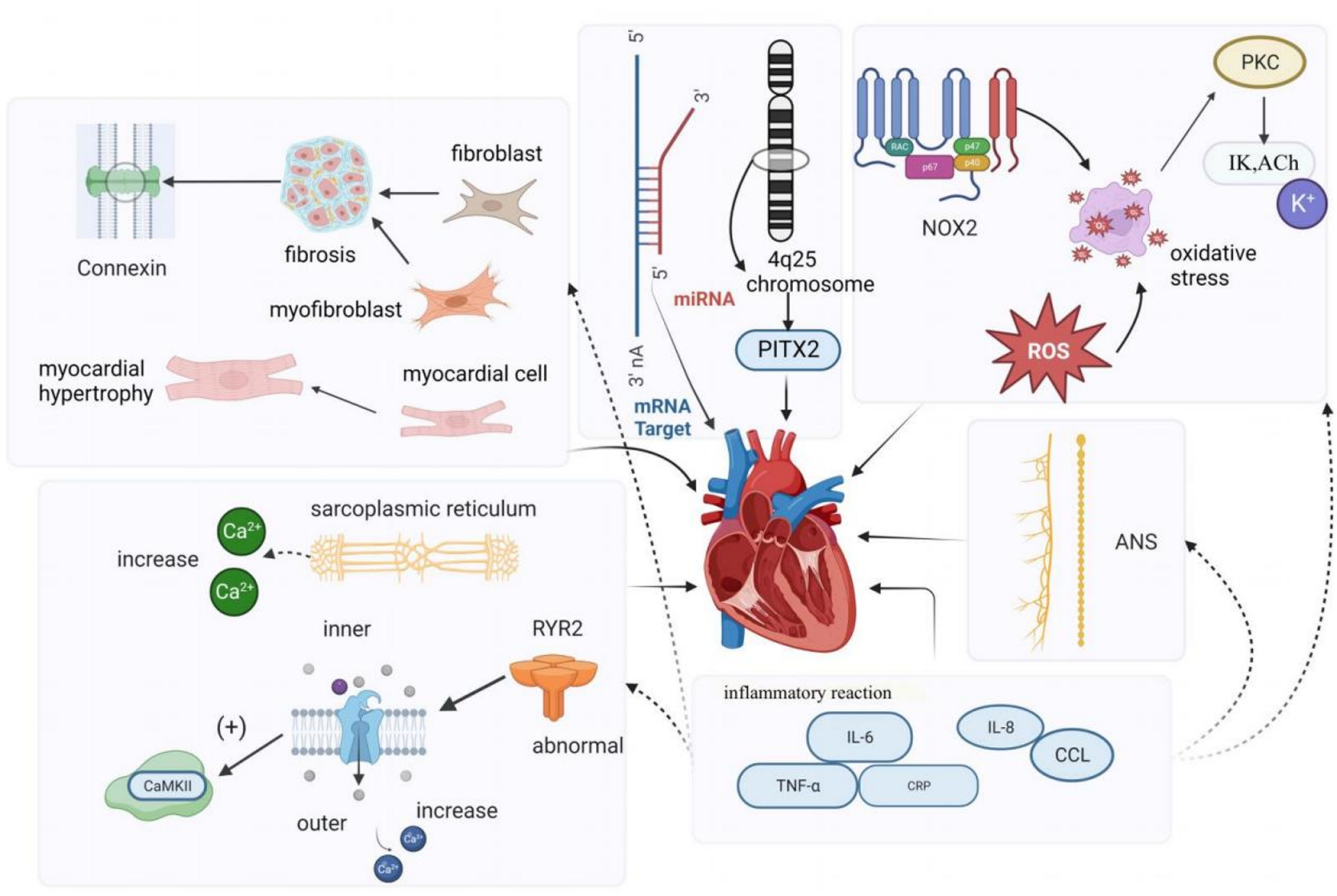
Simple mechanism of KAF.

Early studies have indicated that a family history of AF is linked to a 70% increased risk of AF in offspring (48). Currently, AF genetics is an emerging focus in AF research (49), and microRNAs represent a significant target in genetic studies of AF-related genes that regulate cardiac electrophysiology, as well as the electrical and structural remodeling associated with AF. MicroRNAs, often referred to as miRNAs, are highly conserved noncoding RNAs that are abundantly present in microvesicles and exert control over gene expression at the transcriptional or post-transcriptional level. Some studies have unveiled the crucial role of miRNAs in the development and progression of cardiovascular diseases, including AF. To date, several miRNAs found in atrial tissue, such as miR-1, miR-21, miR-26, miR-29, miR-30a/b, miR-31, miR-328, and miR-208a/b, have been reported to be involved in atrial electrical and structural remodeling (50). Certain studies have identified gain-of-function and loss-of-function mutations in K-channel genes within families exhibiting rare “isolated” AF. Furthermore, in genome-wide association studies involving patients with “isolated” AF, common variants in genes associated with potassium (K) currents have been identified (51). Additionally, genome-wide association studies have identified the chromosome 4q25 locus as the most significant genome-wide association study locus influencing AF susceptibility in the general population to date. Several haplotypes on chromosome 4q25 have been independently linked to an increased risk of AF (52) (Refer to Figure 1).

## 3. Potential mechanisms of AFDAS

Recent studies have revealed that AFDAS represents a distinct clinical condition compared to KAF (53, 54). Approximately 5% of patients who had no previous history of AF may develop AF following an acute ischemic stroke. This newly occurring AF is referred to as newly detected AF or newly diagnosed AF and is typically characterized by being paroxysmal, brief in duration, and asymptomatic (55). The concept of AFDAS has recently been incorporated into the 2020 European Society of Cardiology guidelines for the diagnosis and management of AF (56).

Early detection of AFDAS following a stroke poses a diagnostic challenge, requiring extended electrocardiogram (ECG) monitoring and long-term ECG examination during the initial stages of stroke patients (53, 55). Studies have indicated that AF can be newly diagnosed in around 7%–10% of patients within the first 3–5 days after a stroke. The detection rate increases to 24% or more after cardiac monitoring spanning 6–12 months (7). It’s worth noting that the early-detected symptoms of atrial fibrillation may not necessarily represent newly developed atrial fibrillation after a stroke; they could also signify pre-existing (yet asymptomatic) primary atrial fibrillation (5, 57, 58). Current research has attributed AFDAS to cardiogenic (primary), neurogenic (secondary), and mixed variants. Although the question of whether some AFDAS cases are neurogenic remains subject to debate, clinical evidence still supports the existence of a post-stroke neurogenic mechanism in patients with AFDAS.

In certain instances, AFDAS discovered following an acute ischemic stroke may be temporary and possibly intermittent, resulting from autonomic dysfunction and an immune-inflammatory response triggered by the stroke (6). The majority of recent studies concur that autonomic dysfunction and the immune-inflammatory response are the primary underlying mechanisms of AFDAS. These mechanisms are intricately connected to the insula region of the brain.

### 3.1. Autonomic nervous system

The central autonomic nervous system comprises neuronal groups within various cortical regions, including the insula, ventral medial prefrontal cortex, and anterior cingulate cortex, as well as subcortical regions like the amygdala and hypothalamus, along with the brainstem, including areas like the periaqueductal gray matter of the midbrain, the parabrachial nucleus, the Kölliker-Fuse region of the lateral pons, the solitary bundle nucleus in the medulla, the ventral lateral medulla oblongata, and the intermediate medullary reticular area. These structures regulate cardiovascular function via sympathetic preganglionic and parasympathetic postganglionic fibers collectively known as the exogenous cardiac nervous system. This system connects to the intrinsic cardiac nervous system, consisting of cardiac neurons located within the ganglion plexus found in peripulmonary venous adipose tissue. This intricate network receives impulses from the myocardium and pressure receptors, allowing for autonomic adjustments. Stroke-cardiac syndrome's diverse clinical manifestations are believed to stem from stroke-induced changes in the central autonomic network's function and structure, resulting in dysregulation of cardiac autonomic control. Notably, within the central autonomic network, which encompasses various brain structures, the insular cortex, prefrontal cortex, cingulate cortex, amygdala, hypothalamus, and hippocampus play vital roles in regulating cardiovascular function by modulating sympathetic input to the heart (7). The insular cortex, in particular, is a complex and highly interconnected structure with diverse functions, including interoception, multimodal sensory processing, autonomic regulation, and emotional guidance of self-awareness and social behavior. Research by Seifert et al. (59) suggests that reduced heart rate variability (HRV) indicators are more pronounced in patients with right insula involvement. This implies that the right insular cortex, right frontal cortex, right parietal cortex, as well as lesions in the right amygdala, basal ganglia, thalamus, and their proximity to the development of arrhythmias, are interconnected. Experimental studies have demonstrated that stimulating the left insular cortex tends to evoke a parasympathetic cardiac response, while stimulating the right insular cortex leads to a sympathetic response. Cerebrovascular lesions near the right insular cortex significantly impact heart function (60–62). According to Sposato et al. (11), the autonomic regulation of cardiac rhythm constitutes an integrated transmission system, with the highest control center residing in the cerebral cortex, particularly within the insular region. The occurrence of AF may result from an imbalance between sympathetic and parasympathetic activity, a common consequence following insula infarction, disrupting autonomic regulation and the brain's control of the heart's intrinsic autonomic nervous system. Likewise, Cerasuolo et al. (58) posited, based on heart neuroanatomy, that the heart’s intrinsic autonomic nervous system consists of a ganglia plexus distributed along the ends of the pulmonary veins in the left atrium and within the pericardium. This system is highly regulated by the external autonomic nervous system. Therefore, damage to the insular cortex or its projection areas may trigger AFDAS within the ganglion plexus. Clinical studies conducted by Romano et al. (63) have revealed the critical role of the right insular cortex in autonomic control of cardiac function. Strokes confined to this region can lead to alterations in sympathetic balance. Hilz et al. (64) investigated the relationship between NIHSS scores and autonomic function, concluding that increased stroke severity corresponds to a progressive loss of overall autonomic regulation, reduced parasympathetic tone, heightened stress reflex sensitivity, and a gradual shift toward sympathetic dominance. Such changes within the autonomic nervous system increase the risk of cardiovascular complications and worsen prognosis in severe stroke patients. Additionally, Wang et al. (65) demonstrated that sympathetic overactivity following stroke results in a massive release of catecholamines, which directly overstimulate β-adrenergic receptors on cardiac nerves. This leads to abnormal Ca2+ handling in cardiomyocytes, triggering ectopic cardiac activity. Dorrance et al. (66) also highlighted the role of cerebral ischemia in the release of systemic catecholamines, leading to increased sympathetic tone. The surge in catecholamines overactivates *β* 1-adrenergic receptors, causing intracellular calcium overload, muscle rigidity, metabolic imbalance, and cell death. Furthermore, it overactivates *α* 1-adrenergic receptors, leading to coronary artery constriction and reduced myocardial blood flow.

### 3.2. Immuno-inflammatory response

In the acute phase of a stroke, brain injury triggers a localized inflammatory response, characterized by the proliferation of microglia and astrogliosis. This leads to a substantial release of cytokines and chemokines. Simultaneously, due to damage to endothelial cells, the blood-brain barrier (BBB) becomes more permeable, allowing pro-inflammatory molecules to enter the peripheral circulation through the compromised BBB. This process induces systemic inflammation (67).

It has been demonstrated that inflammatory mediators progressively alter atrial electrophysiology and structural substrates through various signaling pathways, increasing susceptibility to AFDAS (65). Research by Cerasuolo et al. has emphasized the critical role of systemic inflammation in AFDAS development, primarily through the autonomic cascade response and atrial myocarditis. The autonomic cascade response within the ganglion plexus may result from autonomic dysfunction caused by systemic inflammation following a stroke. Prolonged inflammatory responses can lead to atrial remodeling and perpetuate the effects of AF through atrial myocarditis, further contributing to the pro-thrombotic state associated with AF. Furthermore, Sposato et al. (6) have suggested that inflammation triggering AF in the first days after an ischemic stroke may occur through the stimulation of inflammatory mediators on the intrinsic autonomic nervous system and direct damage to the atrial myocardium. Additionally, a study by Victoria et al. (68) has indicated that ischemic stroke patients with localized damage to the right insular cortex develop left atrial coronary microvascular endothelial dysfunction (CMED), myocarditis infiltration (MII), and fibrosis. Even 28 days after a stroke, left ventricular tissue exhibited prolonged fibrosis and B-lymphocyte infiltration. This supports Balint et al.'s findings (69), suggesting that CMED, MII, and fibrosis due to ischemic stroke in the left and right insula are pathological markers of arrhythmogenesis.

Various inflammatory cytokines can significantly affect changes in ion channel function and structural substrates. Studies by Liew et al. have shown that TNF-α overexpression leads to abnormal Ca2+ handling and electrical remodeling. Saba et al. (70) have demonstrated that TNF-α activation also stimulates mouse cardiac fibroblasts and increases matrix metalloproteinase two secretion through the transforming growth factor *β* signaling pathway. IL-6 promotes the synthesis of various inflammatory factors, including TNF-α, C-reactive protein, fibrinogen, and IL-1β, while also promoting ICAM-1 expression and activating matrix metalloproteinases in cardiac myocytes. This induces apoptosis and fibrosis (65). In mouse models, Liao et al. (71) have identified mast cells as critical mediators of allergic and immune responses, which are essential in the pathogenesis of AF in stressed mouse hearts. In contrast, platelet-derived growth factor (PDGF), primarily produced by fibroblasts and mast cells, is another inflammatory cytokine that affects both electrical and structural alterations in cardiac myocytes. PDGF-A promotes cell proliferation and collagen expression in mouse cardiac fibroblasts, leading to atrial fibrosis, increased susceptibility to AF, and electrical remodeling.

**Figure 2:**
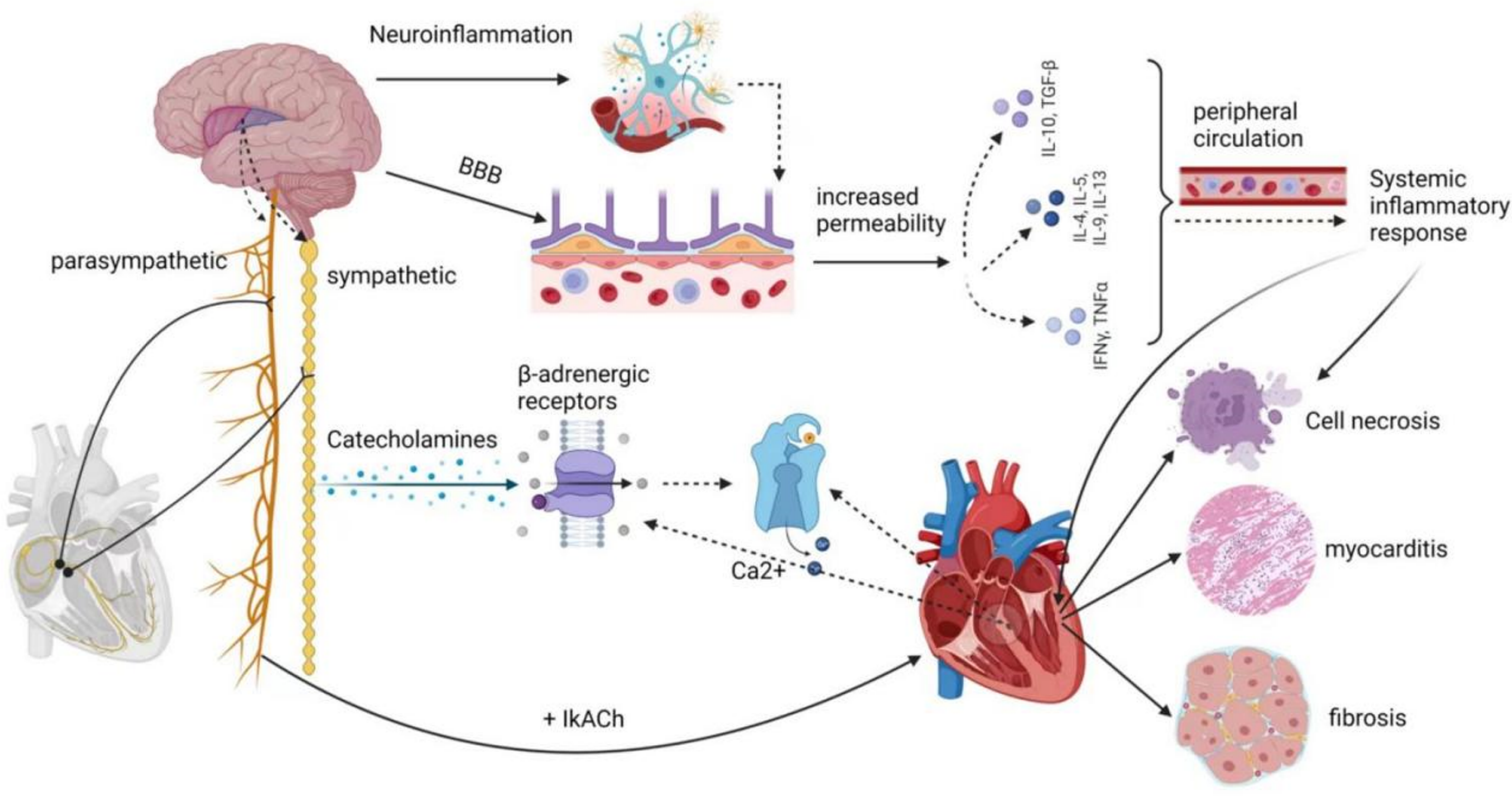
Potential mechanisms of AFDAS.

In summary, current research into the potential mechanisms of AFDAS has centered on the role of autonomic dysfunction, especially after insular damage. The inflammatory response in the heart is triggered by cerebral ischemia, although these mechanistic theories are still in the exploratory research stage. There is no definitive evidence to fully explain the pathophysiological mechanisms of AFDAS. However, the clinical incidence of AFDAS is rising, with studies suggesting that AF may be newly detected in nearly a quarter of stroke or transient ischemic attack patients and in around 5% of patients with no prior history of AF (55, 72). Paroxysmal AF is more common than persistent AF (73). AFDAS increases the risk of recurrent stroke, and mortality rates are 1.5 times higher in patients with AFDAS after a stroke compared to those without AF (74). According to current guidelines, oral anticoagulants are the preferred choice for early prevention and treatment of AFDAS. However, anticoagulants pose a bleeding risk and may not be suitable, especially for patients with hemorrhagic strokes. Therefore, the need for new alternative treatments for early prevention and management of AFDAS is pressing (Refer to Figure 2).

In this article, we have broadened the concept of KAF to encompass cardiogenic AF resulting from underlying cardiac abnormalities. This type of AF not only significantly elevates the risk of thromboembolism but is also associated with a higher prevalence of coronary artery disease, congestive heart failure, prior myocardial infarction, and a history of cerebrovascular events compared to AFDAS. Patients with KAF typically exhibit larger left atrial dimensions and lower left ventricular ejection fractions than those with AFDAS. Moreover, patients with KAF face a heightened risk of stroke recurrence compared to AFDAS patients. It’s worth noting that in clinical practice, there is a subtype of atrial fibrillation known as secondary atrial fibrillation (SAF), which shares some similarities with AFDAS. SAF is characterized by self-limiting and reversible clinical features and is often secondary to conditions such as surgery, acute infections, and myocardial infarction. Postoperative atrial fibrillation (POAF) is the most common form of SAF, especially following valve replacement surgery. Inflammation and the influence of the autonomic nervous system are key mechanisms in the development of POAF, somewhat resembling the potential mechanisms in AFDAS (75). Some studies suggest that SAF patients may have different stroke and bleeding risks compared to KAF patients. SAF patients have a higher bleeding risk than KAF patients, and this bleeding risk may even surpass their risk of stroke. This conclusion is drawn from observations that the use of anticoagulants does not effectively reduce the risk of ischemic stroke in newly diagnosed atrial fibrillation patients associated with acute coronary syndrome (ACS), acute pulmonary disease, and sepsis. Furthermore, a higher bleeding risk has been noted in patients with acute pulmonary disease, leading to the conclusion that anticoagulation therapy's benefits for SAF may be limited, offering no advantage in reducing stroke risk and potentially increasing the risk of bleeding (76). Additionally, research has shown that secondary atrial fibrillation patients face a lower average recurrence risk and a reduced risk of heart failure. However, this does not imply that we can disregard the risk of recurrence. Infection-induced secondary atrial fibrillation carries a thromboembolic risk twice as high as non-infection-induced atrial fibrillation and is associated with higher mortality and poorer treatment outcomes (77). For newly diagnosed and secondary atrial fibrillation patients, heightened vigilance for recurrent atrial fibrillation may be necessary. Nonetheless, the optimal monitoring and thromboembolic prevention strategies for SAF patients remain unclear at this time (78).

In summary, KAF, SAF, and AFDAS all pose significant clinical challenges in contemporary medicine, and early imaging, such as head CT or MRI, long-term electrocardiogram, cardiac monitoring, or Holter testing, should be conducted for early prevention and treatment.

## 4. Current therapeutic advances and limitations

Over the past decade, significant breakthroughs have occurred in understanding atrial fibrillation (AF), encompassing epidemiology, genetics, electrophysiology, and molecular cell biology. Concurrently, there has been a growing focus on research related to brain-cardiac syndrome and stroke-cardiac syndrome, strengthening the connection between brain function and cardiac health. This has accentuated the need to urgently address the clinical issues surrounding cardiac complications arising from strokes. In the 2020 guidelines by the European Society of Cardiology (ESC) for the diagnosis and treatment of AF, patients with AF are recommended to undergo a thorough evaluation based on four key criteria. These criteria encompass assessing stroke risk using the CHA2DS2VASc score, evaluating symptom severity with the EHRA Score, gauging the severity of AF burden (which includes self-termination, paroxysmal, persistent, and permanent forms), and assessing substrate severity, which takes into account factors like aging, comorbidities, and structural heart disease. These guidelines also propose a comprehensive A-B-C approach to treatment.

### 4.1. Stroke avoidance

In this context, “A” represents stroke avoidance, highlighting the role of anticoagulation therapy. While current clinical practice recommends oral anticoagulants as the primary treatment for AFDAS, it’s important to note that the use of oral anticoagulants has been associated with an increased risk of bleeding, recurrent ischemic stroke, and mortality (79). Although the adverse risks associated with early anticoagulant use are lower compared to later stages of treatment (80), it’s crucial to understand that this form of anticoagulation primarily aims to prevent and manage complications related to ischemic strokes rather than addressing the root cause of the condition.

### 4.2. Better symptom management

“B” represents better symptom management, emphasizing heart rate and rhythm control. Early rhythm control refers to prompt intervention with antiarrhythmic medications or AF ablation for patients with early atrial fibrillation and underlying cardiovascular diseases. A growing body of evidence supports the effectiveness of early rhythm control in high-risk patients, as it can reduce irreversible atrial remodeling, prevent adverse outcomes like AF-associated death, heart failure, and stroke, and potentially halt the progression of AF, sparing patients from years of symptomatic AF (80–82). Current clinical treatments for KAF involve both pharmacologic and non-pharmacologic approaches. First-line pharmacologic therapy primarily includes antiarrhythmic drugs, with amiodarone being one of the most commonly used and effective antiarrhythmic drugs. These drugs primarily target ion channels, although their efficacy in maintaining normal heart rhythm, especially as first-line drugs, is somewhat limited. Additionally, they come with various toxicity concerns (83), such as amiodarone, which can potentially lead to thyroid dysfunction, pulmonary fibrosis, skin disorders, and other minor adverse reactions (84). Some antiarrhythmic drugs even have proarrhythmic effects and an increased risk of death (85, 86).

Apart from antiarrhythmic drugs, modern clinical studies have introduced various innovative ideas and methods for AF treatment. For example, research by Wiedmann investigated the effectiveness of Doxapram in antiarrhythmic effects using large clinical animal models. They based their study on the significant upregulation of TASK-1 (K2P3.1) in AF patients, providing a basis for inducing AF-related electrical remodeling. The study found that Doxapram could block the upregulation of atrial TASK-1 current and associated shortening of the action potential duration (APD), successfully inducing acute cardioversion in paroxysmal AF and rhythm control in persistent AF. This experiment served as a preclinical pilot study, demonstrating Doxapram's potential as a Class III antiarrhythmic agent, with further clinical trials needed to assess its impact on AF patients (87). Simultaneously, in another study, they also discovered that the TASK-1 inhibitor A293 could induce cardiac cardioversion in paroxysmal and persistent AF pig models, exhibiting antiarrhythmic effects (88, 89). Currently, the K2P3.1 channel has emerged as a novel strategy for treating atrial fibrillation. However, it’s worth noting that some research indicates an association between the new gene KCNK3 and familial and idiopathic pulmonary arterial hypertension, potentially elevating pulmonary artery pressure (89, 90).

SK channels have also become a new target for AF treatment, similar to TASK-1, primarily expressed in the atria. Studies suggest that in the late phase of inducing atrial fibrillation action potentials, SK channels contribute to repolarization of atrial myocardial cells. By blocking SK channels, one can prolong the action potential duration of atrial myocardial cells, reduce their excitability, and aid in restoring normal rhythm (91). Diness and their team explored a novel SK channel inhibitor—AP14145 in their research. They demonstrated that AP14145 selectively prolonged the refractory period of the pig left atrium and shortened the duration of acutely induced AF, but it’s important to note potential adverse effects such as vomiting. In another study, they evaluated the antiarrhythmic effects of three SK channel inhibitors, UCL1684, N-(pyridin-2-yl)-4-(pyridin-2-yl)thiazol-2-amine (ICA), and NS8593. These three drugs were found to prolong atrial effective refractory period without affecting the QT interval, effectively preventing or terminating AF, and were equally suitable for patients with paroxysmal AF and hypertension (92–94). However, SK channel blockers still need further testing in large-scale phase III trials.

The renin-angiotensin-aldosterone system (RAAS) plays a role in the development and progression of AF, with angiotensin II activating intracellular signaling cascades that lead to cardiomyocyte hypertrophy, apoptosis, and fibroblast proliferation. Li et al. (95) demonstrated that sacubitril/valsartan could improve atrial remodeling and ultimately reduce the occurrence and recurrence of AF by inhibiting RAAS activation and lowering blood pressure. However, this type of drug may be more targeted to patients with hypertension-induced AF, and its therapeutic scope may be relatively limited. Recent studies have suggested that elevated expression of Hsp27, a chaperone shock protein, observed in patients with paroxysmal AF, may help protect cardiomyocytes and limit the progression of persistent AF (96). Polyglutinyl acetone (GGA), a natural product extracted from licorice, is thought to have various biological activities such as anti-inflammatory, antioxidant, and anti-stress properties. Some studies have found that HSP induction can prevent remodeling induced by atrial tachycardia. Oral HSP inducer GGA has been shown to prevent AF in clinically relevant animal models, suggesting the use of HSP inducers as a novel approach to treating atrial fibrillation (97). Chloroquine, an anti-malarial drug, has shown promise in treating AF as it selectively blocks inwardly rectifying K+ channels (98–100). However, prolonged use of chloroquine is not without systemic effects. Vericiguat, a soluble guanylate cyclase agonist, has been investigated for its potential to treat AF by decreasing electrical and structural remodeling in rabbit models of AF. However, the specific mechanism of its effect on AF remains unclear, and the drug has not yet been widely validated, although it may hold potential value in AF treatment (101). Other studies have indicated that endoplasmic reticulum stress-induced autophagy is a crucial pathway in the progression of AF. Experimental evidence has shown that 4-phenylbutyrate can prevent electrical remodeling and slow the progression of AF by inhibiting autophagy activation and transient calcium loss, highlighting the potential therapeutic benefits of the endoplasmic reticulum stress inhibitor, 4-phenylbutyrate (102). Zhang et al. also suggested that histone deacetylase (HDAC6) has the potential to prevent AF-associated remodeling, indicating that HDAC6 could be a viable therapeutic target for clinical AF treatment. Ying et al. (103) demonstrated that colchicine could reduce AF recurrence by mitigating electrical remodeling in post-surgical AF. This effect is achieved through the inhibition of immune-related gene expression and the stabilization of microtubules. “IKur,” an ultra-rapid delayed-rectifier K+ current, is exclusive to the atria, and several drugs have shown that effective IKur inhibition can terminate AF and prevent its recurrence (104). However, highly selective IKur blockers are not effective in prolonging atrial refractoriness and are downregulated in the human atria during persistent atrial fibrillation, raising questions about the relevance of IKur as a target for anti-fibrillation therapies (105, 106). A relatively low dose of galactomannan (GM CT-01), administered intravenously, demonstrated the ability to reduce both structural and electrical remodeling, as well as the burden of AF in a sheep model of persistent AF without comorbidities. However, it’s important to note that Gal-3 inhibition did not lead to the long-term restoration of sinus rhythm (105). Among the protein family, Cx40 appears to be one of the most promising therapeutic targets because of its high expression in the atria while not being present in the ventricles. Shiroshita-Takeshita et al. (107) discovered that antiarrhythmic peptides improved gap-function conduction, with rotigaptide showing improved conduction velocity in several animal models. Although antiarrhythmic peptides hold potential benefits for AF, their effects may not be consistent throughout the atrium, possibly exacerbating the existing heterogeneity of atrial electrophysiology. This interaction could be influenced by factors such as fibrosis and ion channel remodeling in the atria. The G-protein-IP3-Ca2+ signaling axis has also been proposed as a potential target for AF treatment, acting by modulating intracellular calcium ion concentrations (108). However, doubts remain regarding its selectivity, as this axis is functionally active in many cellular processes and cell types, which could lead to potential adverse events in the heart and other organs. Consequently, no drugs targeting the G-protein-IP3-Ca2+ signaling axis have been tested for atrial fibrillation treatment in humans (105). Inflammation of atrial tissue is implicated in arrhythmic remodeling and is considered a potential target for antiarrhythmic therapy. Corticosteroids, known for their potent anti-inflammatory effects, have been studied for their ability to prevent atrial fibrillation after cardiac surgery (109). However, their use is limited due to potential adverse effects, especially with long-term use.

In addition to Western medicine, traditional Chinese herbs have been explored for AF treatment. WenXin Granules, a Chinese medicinal preparation consisting of various herbs like Gan Song, Codonopsis, Panax Ginseng, Succinum, and Rhizoma Polygonati Odorati, has been used to aid in the treatment of cardiovascular diseases. It offers benefits such as improving blood circulation, reducing blood stasis, dilating blood vessels, and regulating cardiac function. Research has suggested that WenXin Granules might exhibit atrial-selective inhibitory effects on INa (110).

In addition to drug therapy, non-pharmacological treatments primarily encompass the following methods: electrical cardioversion, pulmonary vein electrical isolation, ablation, left atrial appendage occlusion, gene therapy, pacemaker implantation, low-level vagus nerve stimulation, and acupuncture. It’s worth noting that His bundle ablation, cardiac pacing, and electrical cardioversion do not prevent the occurrence and progression of new AF and necessitate continued anticoagulation therapy. His bundle ablation, which disrupts the His bundle in the conduction system between the atria and ventricles to block abnormal electrical signaling and restore normal rhythm, traditionally requires continuous right ventricular pacing after interrupting ventricular conduction. This can potentially lead to a deterioration of cardiac function. However, modern physiological pacing strategies, such as LBB/LB area pacing, physiological rate-responsive pacing, multi-chamber pacing, and adaptive pacing, have emerged to address the issues associated with adverse reactions seen in traditional pacing. These strategies maintain synchronous contraction of the left and right ventricles, reducing adverse reactions and improving the effectiveness of cardiac treatment and patient quality of life (111). Nonetheless, it’s important to recognize that not all patients may require or benefit from these modern physiological pacing strategies, and factors such as regular monitoring and economic costs also need consideration. Catheter ablation offers better efficacy in rhythm control compared to antiarrhythmic drugs but carries a significant rate of AF recurrence, particularly in patients with persistent AF, which may necessitate repeat procedures (112). There are numerous complications associated with catheter ablation, with vascular complications being the most common, followed by pericardial effusion, pericardial tamponade, and stroke or transient ischemic attack (86). Among the different types of catheter ablation, radiofrequency (RF) ablation is a procedure that disrupts abnormal rhythmic sources or conduction pathways using radiofrequency energy. However, RF ablation can lead to complications such as cardiac perforation, esophageal injury, and pulmonary stenosis. Cryoballoon ablation, which freezes abnormal rhythm sources or conduction pathways, can result in complications like phrenic nerve injury (113). In contrast, pulsed field ablation (PFA) is a novel non-thermal ablation method that selectively ablates cardiac tissue while preserving other anatomical structures, thus avoiding complications associated with thermal ablation (114). Studies have shown PFA to be a feasible and safe ablation method for mitral isthmus ablation in persistent atrial fibrillation patients, in addition to pulmonary vein isolation (PVI). However, some reversible and non-lethal adverse events were observed in experiments, such as coronary artery spasm and a 20% recurrence rate (115). Furthermore, studies comparing cell death induction between radiofrequency ablation, cryoballoon ablation, and PFA ablation have suggested that PFA ablation may offer faster, safer, and more tissue-selective ablation with less inflammation due to apoptosis-dominated cell death (116).

**Figure 3:**
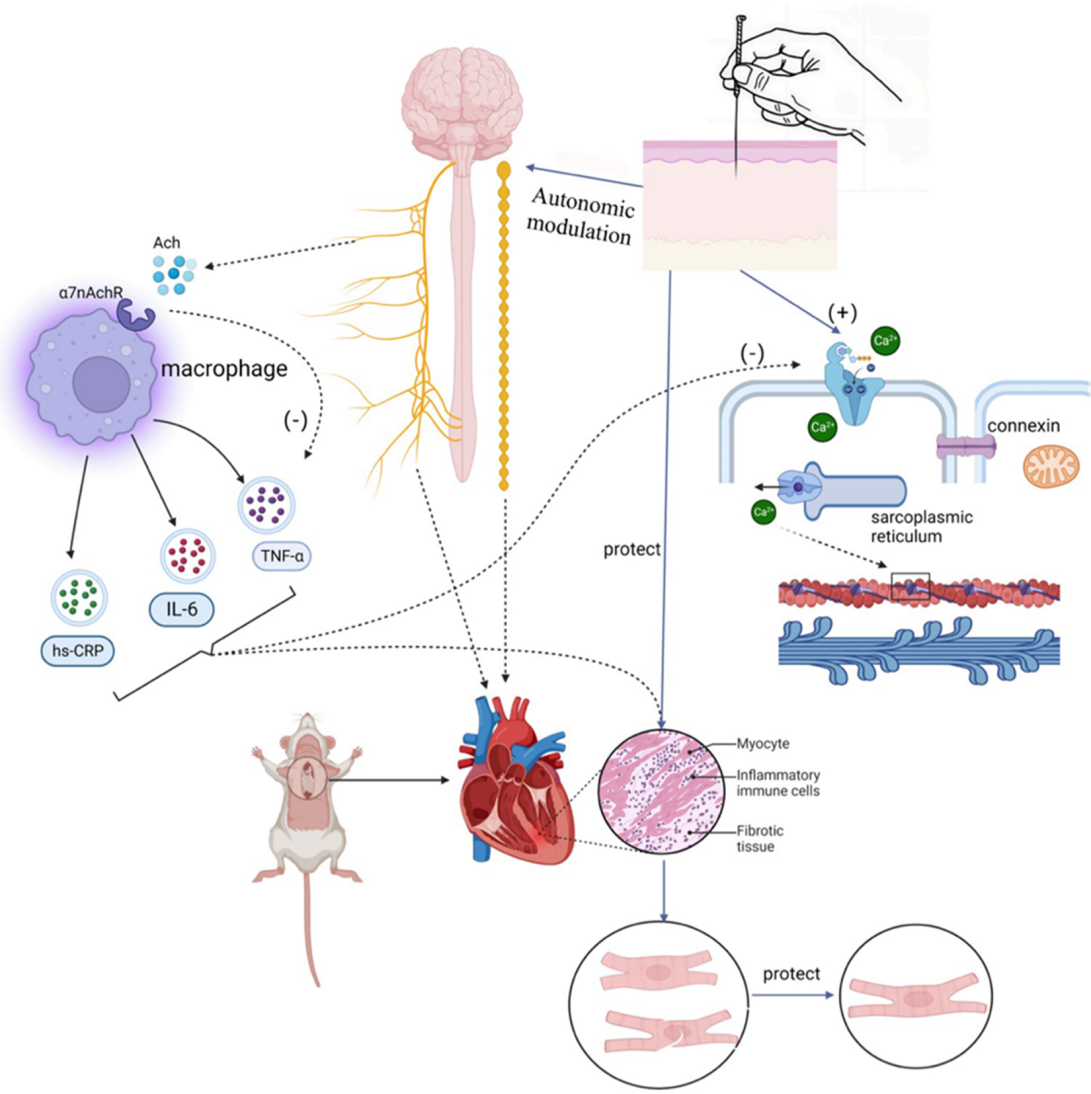
Mechanism hypotheses diagram of autonomic modulation in AF.

Left atrial appendage occlusion is a surgical treatment used to prevent AF-related thromboembolism. It does not directly target AF but rather avoids the potential risks and side effects associated with anticoagulants. However, this procedure is indicated for specific groups of patients, such as those at high risk of embolism, those unable to tolerate anticoagulant medications, or those experiencing complications from anticoagulant therapy. Gene therapy for AF is an emerging therapeutic approach aimed at treating or alleviating AF symptoms by modulating or repairing genetic variations associated with AF. These approaches include gene repair, gene-targeted therapy, and gene modulation. While preliminary studies and clinical trials have shown potential therapeutic effects, gene therapy is still in the research and experimental stage in the context of AF treatment. More studies and clinical trials are needed to determine its safety and efficacy before clinical application. Autonomic activity has been found to play a significant role in initiating and maintaining AF (23). Modulating autonomic function may help control AF, suggesting potential therapeutic tools such as ganglion plexus ablation, renal sympathetic denervation, cervical vagus nerve stimulation, pressure reflex stimulation, skin stimulation, novel pharmacological approaches, and biological therapies (23). Low-level vagus nerve stimulation is a recently introduced treatment for AF that involves stimulating the dominant auricular branch of the vagus nerve, located in the external acoustic canal and the skin of the auricle. It has been effective in reducing the incidence and burden of AF (117, 118). Studies have shown that low-level vagal nerve stimulation can help suppress paroxysmal AF by attenuating the inflammatory response through the activation of cholinergic anti-inflammatory pathways (119). Additionally, sustained low-level vagal nerve stimulation has been shown to suppress stellate ganglionic neural activity and reduce the incidence of paroxysmal atrial tachyarrhythmias (120). Acupuncture, as an effective non-invasive and safe treatment tool, has demonstrated the ability to significantly reduce the number and duration of symptomatic AF attacks. It also has a beneficial effect on maintaining sinus rhythm and converting AF to sinus rhythm in acute cases (121). Moreover, acupuncture has been shown to prevent recurrence in patients with persistent AF after cardioversion (122, 123). One of the mechanisms behind its effectiveness lies in the regulation of autonomic function. Based on current research, the effectiveness and potential mechanisms of acupuncture in AF treatment can be categorized into four aspects: regulating anti-inflammatory factors, modulating ion channels and connexins, regulating the autonomic nervous system, and enhancing the ultrastructure of atrial muscle (124–126) (Refer to Figure 3).

### 4.3. Cardiovascular risk factor and comorbidity management

**Figure 4:**
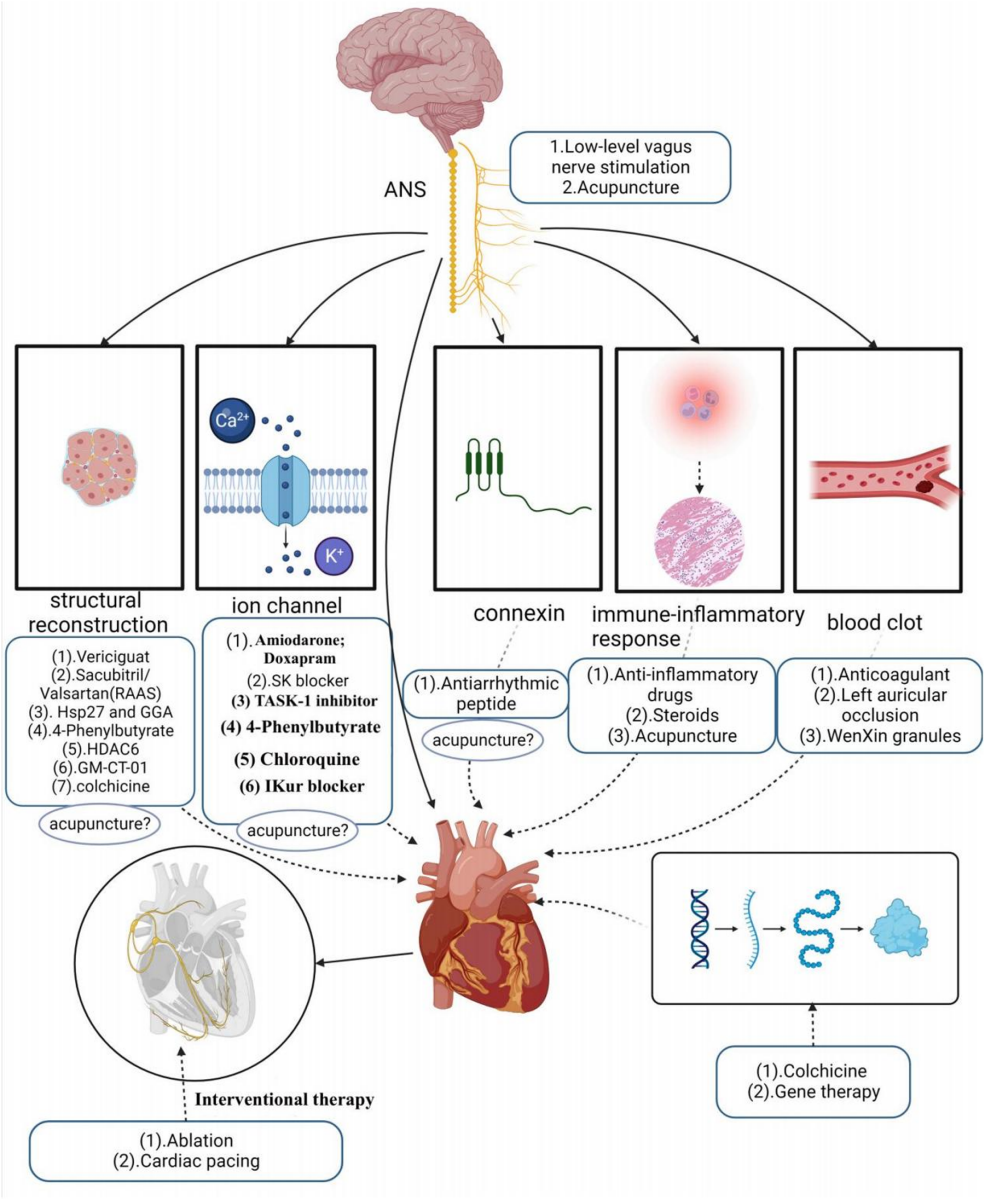
Clinical therapeutic mechanisms diagram.

“C” represents cardiovascular risk factor and comorbidity management, emphasizing the importance of early preventive management of AF and its complications. This management primarily includes lifestyle changes, nutritional health treatment. Lifestyle changes entail adopting moderate exercise, improving physical fitness, and increasing exercise training, all of which can reduce the recurrence of AF and provide overall cardiovascular benefits. However, it’s worth noting that intense exercise may elevate the risk of developing atrial fibrillation in individuals with the condition (127). Research has shown that both physical and mental exercises, such as yoga, tai chi, and qigong, have favorable effects on cardiac autonomic function, may alleviate symptoms in AF patients, normalize biomarkers of AF, and promote healthy aging. Nutritional therapy involves making dietary adjustments, ensuring balanced nutrition, and following a low-fat diet, all of which can help reduce the accumulation of body and visceral fat (128). Obesity is associated with an increased risk of developing AF (129), so strategies to avoid excessive weight gain are essential for preventing AF and its recurrences after the first episode. These measures also contribute to improving patient symptoms and enhancing their overall quality of life (130) (Refer to Figure 4).

## 5. Prospects and outlook

The development of AF is intricate, and it may involve multiple factors. Therefore, a single pathway of drug therapy may be suitable for some patients but not for everyone. While the A-B-C pathway of integrated care may help compensate for the limitations of a single pathway, reduce some risks, and achieve a comprehensive and holistic approach to preventive treatment, most therapeutic interventions within each pathway still predominantly target the symptoms or single mechanisms of AF. Furthermore, the majority of therapeutic goals are directed toward downstream pathways, with limited impact on upstream pathways. We should contemplate how to predict new treatment strategies based on the diversity of AF mechanisms and upstream pathways.

Current research suggests that mechanism-based therapies for AF are still in their early stages, and the strong connection between the brain and the heart is gaining prominence as brain-heart syndrome research expands (7, 131). In this discussion, when summarizing the entire pathogenesis of AF briefly, it becomes apparent that the central nervous system, housing the brain and autonomic nerves, plays a pivotal role in AF development. It not only directly regulates the heart but also acts as an upstream pathway influencing downstream pathways like cardiac structural remodeling, immune-inflammatory responses, ion channels and connexins, vasoconstriction, and thrombosis, ultimately affecting cardiac function. However, most current therapeutic approaches primarily target the downstream pathways and lack consideration for the central nervous system and upstream pathways. This limitation narrows the scope of action and increases the likelihood of recurrence. As our understanding of AF genetics, the brain-heart axis, vagus nerve stimulation therapy, and autonomic function modulation continues to grow, the range of targets for AF treatment will likely expand. It may be possible to effectively treat AF by focusing on the upstream pathway to guide the coordinated action of multiple downstream pathways. Predictably, central nervous system regulation will likely become a major focus of AF treatment in the future. However, this process will require extensive experimental studies and clinical validation to progress. With advancements in technology and increased comprehensive mechanistic exploration and integrated research efforts, we can gradually unravel the diversity and dynamic changes in AF mechanisms. This will pave the way for innovative approaches in both pharmaceutical and non-pharmaceutical treatments. While translating these novel mechanism-based therapeutic strategies into clinical applications poses significant challenges and demands interdisciplinary, large-scale, and sustained collaborative efforts, we maintain optimism about the future of atrial fibrillation treatment and research based on the current landscape.
